# Systemic air embolism causing acute stroke and myocardial infarction after percutaneous transthoracic lung biopsy—a case report

**DOI:** 10.1186/s13019-015-0329-3

**Published:** 2015-09-15

**Authors:** Wei-Heng Hung, Chun-Chi Chang, Shang-Yun Ho, Chiung-Ying Liao, Bing-Yen Wang

**Affiliations:** 1 Division of Thoracic Surgery, Department of Surgery, Changhua Christian Hospital, No. 135, Nanxium St, Changhua City, Changhua County 500 Taiwan; 2 Division of Chest Medicine, Department of Internal Medicine, Changhua Christian Hospital, Changhua, Taiwan; 3 Department of Medical Imaging, Changhua Christian Hospital, Changhua, Taiwan; 4 Division of Thoracic Surgery, Department of Surgery, Taipei Veterans General Hospital Taipei, and School of Medicine, National Yang-Ming University, Taipei, Taiwan; 5 Institutes of Medicine, Chung Shan Medical University, Taichung, Taiwan

**Keywords:** Air embolism, Biopsy, Stroke, Myocardial infarction

## Abstract

Computed tomography (CT)-guided transthoracic lung biopsy is a common procedure for the diagnosis of pulmonary lesion. Pneumothorax, pulmonary hemorrhage and hemoptysis are the most common complications of the procedure. Air embolism is a rare serious complication. We reported a case with air embolism related acute ischemic stroke and non-ST elevation myocardial infarction (NSTEMI) simultaneously after percutaneous transthoracic lung biopsy.

## Background

Computed tomography (CT)-guided transthoracic lung biopsy is a common procedure for the diagnosis of pulmonary lesion. The procedure is usually safe but still not free of complications. Pneumothorax, pulmonary hemorrhage and hemoptysis are the most common complications. The serious complications such as air embolism sometimes occurred. The incidence has been reported to be 0.02 % to 0.07 % [1–3]. Air embolism is rare but potentially life-threatening complication of CT-guided transthoracic lung biopsy. Air embolism could induce acute ischemic stroke or acute myocardial infarction and caused patient to death. We reported a case with air embolism related acute ischemic stroke and non-ST elevation myocardial infarction (NSTEMI) simultaneously after CT-guided transthoracic lung biopsy.

## Case presentation

**Fig. 1:**
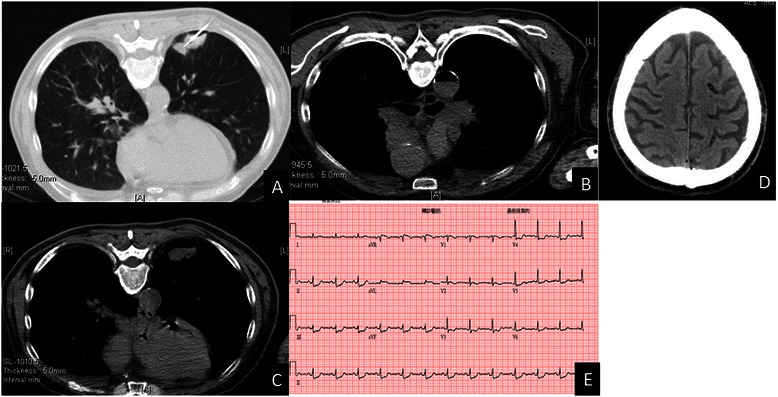
**a** Transthoracic biopsy with 18G coaxial niddle for a pulmonary tumor. **b** Chest computed tomography revealed air-fluid in the aorta. **c** Chest computed tomography showed air bubbles in the coronary artery. **d** Brain computed tomography demonstrated multiple air embolisms in the cerebral vessels. **e** Electrocardiography also showed ST-segment depression in leads II, III and aVF

A 63-year-old man underwent a chest CT-guided biopsy of lung tumor in prone position (Fig. 1a). Subsequent chest CT demonstrated air-fluid in the aorta and air embolism in the coronary artery (Fig. 1b-c). Consciousness disturbance with urine incontinence developed after lung biopsy immediately. Neurological examination revealed right hemiplegia with drowsy consciousness and global aphasia. Brain CT showed multiple air embolisms in the cerebral vessels (Fig. 1e). Post-lung biopsy air emboli related acute ischemic stroke over left middle cerebral artery territory was impressed. Cold sweating developed at the same night. Elevated cardiac enzymes levels were noted. Electrocardiography also showed ST-segment depression in leads II, III and aVF. (Fig. 1d). Air emboli related non-ST elevation myocardial infarction (NSTEMI) was found. Conservative treatment with aspirin used, intravenous fluid hydration and oxygen therapy with FiO2 50 % were administrated. Right hemiplegia improved gradually and cardiac enzymes level also subsided. The patient was discharged smoothly 7 days after lung biopsy.

## Discussion

Air embolism is a rare complication of the CT-guided lung biopsy and is potentially fatal. Air can enter a pulmonary vein branch either directly via the entry needle of a coaxial system or through a fistulous connection (created during the biopsy) between an airway and adjacent pulmonary vein. The air bubble may then pass into the left heart and subsequently occlude to the coronary or cerebral circulation. This could result in myocardial infarction or stroke. The risk of air embolism is increased in the biopsy of more central lung lesions due to the increased diameter of the bronchovascular bundle [4].

Air embolism may be prevented by avoiding needle biopsies of cysts, cavities, or bullous lung parenchyma. In addition, a stylet or occlusion of the hollow needle at all times can prevent direct communication between the atmosphere and pulmonary venous system. The patient should refrain from coughing or straining while the mass is being biopsied, especially when the stylet has been removed. It is crucial to select an insertion site where the needle penetrates the least amount of lung parenchyma to reach the mass. Performing the biopsy under CT-guided fluoroscopy may decrease the incidence of this complication [5]. Freund et al. [6] found the depth of the needle in the lesion (Needle tip not in the tumor is risk factor), endotracheal anesthesia, location of the lesion above the level of the left atrium, and prone position of the patients were independent risk factors for the incidence of a systemic air embolism. In our case, the lesion was under the level of the left atrium. The procedure was performed under local anesthesia and the needle tip was in the tumor. But the patient was positioned in prone position. Prone position could be the risk factor of systemic air embolism in our case.

In previous studies [7–11], hyperbaric oxygen therapy has been considered the primary therapy by reducing bubble volume and improving tissue oxygenation. The size of a gas bubble is inversely proportional to ambient pressure at constant temperature. In our case, the patient did not receive hyperbaric oxygen therapy but other conservative treatment; he was still discharged smoothly 7 days after lung biopsy.

## Conclusion

Although the incidence rate of air embolism is low after CT-guided transthoracic lung biopsy, its potential mortality should be respected.

## Consent

Written informed consent was obtained from the patient for publication of this Case report and any accompanying images. A copy of the written consent is available for review by the Editor-in-Chief of this journal.

## Abbreviations

CT: Computed tomography
NSTEMI: Non-ST elevation myocardial infarction
